# How to Find out If a Case of Gonorrhœa Is Actually Cured

**Published:** 1894-11

**Authors:** 


HOW TO FIND OUT IF A CASE OF GONORRHŒA IS ACTUALLY CURED.—Dr. Kraft (*La Semaine Medicale*, No 49, 1894), of Utrecht, Holland, has a very ingenivs and at the same time efficacious method of testing whether in a given case of gonorrhœa an actual cure has been obtained. As is known, nothing is more difficult than to be able to say whether a gonorrhœa which has ceased to discharge has really and definitely been cured. The cessation of discharge, the absence of gleet and the agglutination of the lips of the meatus are easily absent while the disease is present, though latent, and still virulent enough to be transmitted by coitus. In such cases the absence of these signs may be the means of placing the physician in an embarrassing position. For example, a patient, who has had a gonorrhœa and is about to marry, asks his physician whether he is completely freed from his disease and without danger of contaminating his wife. In such cases the writer has the patient drink a quart and a half of beer, while he injects into the patient's urethra a 2 per cent. solution of sublimate. If he is actually cured no reaction follows; if the contrary is true a discharge will be set up, which sometimes does not appear for forty-eight hours.—*Exchange.*

